# New MoS_2_/Tegafur-Containing Pharmaceutical Formulations for Selective LED-Based Skin Cancer Photo-Chemotherapy

**DOI:** 10.3390/pharmaceutics16030360

**Published:** 2024-03-04

**Authors:** Miguel T. Campos, Filipa A. L. S. Silva, José Ramiro Fernandes, Susana G. Santos, Fernão D. Magalhães, Maria J. Oliveira, Artur M. Pinto

**Affiliations:** 1 LEPABE, Faculdade de Engenharia, Universidade do Porto, 4200-465 Porto, Portugal; migueltcampos1234@gmail.com (M.T.C.); fdmagalh@fe.up.pt (F.D.M.); 2 ALICE—Associate Laboratory in Chemical Engineering, Faculdade de Engenharia, Universidade do Porto, 4200-465 Porto, Portugal; 3 i3S—Instituto de Investigação e Inovação em Saúde, Universidade do Porto, 4200-135 Porto, Portugal; 4 INEB—Instituto de Engenharia Biomédica, Universidade do Porto, 4200-135 Porto, Portugal; 5 CQVR—Centro de Química Vila Real, University of Trás-os-Montes and Alto Douro, 5000-801 Vila Real, Portugal; jraf@utad.pt; 6 Physical Department, University of Trás-os-Montes and Alto Douro, 5000-801 Vila Real, Portugal

**Keywords:** photothermal therapy, 2D nanomaterials, transition metal dichalcogenides (TMDs; TMDCs), biocompatibility, targeted selective therapy, anticancer drugs

## Abstract

Non-melanoma skin cancer (NMSC) is one of the most common types of cancer worldwide. Despite the low mortality rate, rising incidence and recurrence rates are a burden on healthcare systems. Standard treatments such as chemotherapy, radiotherapy, and surgery are either invasive or toxic to healthy tissues; therefore, new, alternative, selective treatments are needed. In this work, a combined photothermal and chemotherapeutic approach is proposed. MoS_2_ was used as photothermal agent. It was prepared by a liquid-phase exfoliation and intercalation method using polyvinylpyrrolidone (PVP), followed by recirculation through a custom-built high-power ultrasonication probe. After 6 h of ultrasonication treatment, the average particle size was 165 ± 170 nm. Near-infrared (NIR) irradiation assays (810 nm, 0.1 W/cm^2^, 30 min, 180 J/cm^2^) confirmed that MoS_2_ nanosheets can efficiently convert NIR light into heat and reach 52 °C. The therapeutic doses of MoS_2_ (125 µg/mL) and Tegafur (50 µg/mL) were optimized and both were simultaneously incorporated into a Carbopol hydrogel. The cells were brought into contact with the hydrogel and irradiated with a custom-built NIR LED system. In HFF-1 cells (normal human fibroblasts), the metabolic activity was 78% (above the 70% toxicity limit—ISO 10993-5:2009(E)), while in A-431 skin cancer cells, it was 28%. In addition, the MoS_2_ + Tegafur hydrogels led to a 1.9-fold decrease in A-431 cancer cell metabolic activity, 72 h after irradiation, in comparison to MoS_2_ hydrogels, indicating a combined effect of photothermal and chemotherapy.

## 1. Introduction

Skin cancer is one of the most common types of cancer worldwide [1,2]. Non-melanoma skin cancer (NMSC) is the most common form of skin cancer and is increasingly common in patients under the age of 40, with basal cell carcinoma accounting for 80% of all NMSC cases [3,4]. The treatments used to cure NMSC are based on the risk that each case poses to the patient’s health [5,6]. The most important therapies include surgery, chemotherapy, and radiotherapy [7]. Despite their frequent use, all of these therapies also have disadvantages [8]. Surgery consists of removing the tumor tissue, although sometimes, complete removal is not achieved, leading to recurrence; also, surgical trauma causes high inflammation and reduces the anticancer immune response [9,10]. Often, the aesthetic results are not ideal, considering that NMSC is more common on the face and neck [11,12]. Chemotherapy can lead to cancer cell resistance to the drugs and local and systemic toxicity to normal cells and tissues [8,13,14]. Radiotherapy can lead to systemic symptoms such as malaise, hair loss, loss of fertility, and indiscriminate cell destruction [8]. Photothermal therapy (PTT) has been investigated as a new therapeutic approach for cancer treatment as it is a non-invasive, cost-effective, and specific therapeutic approach. PTT is based on the irradiation of nanomaterials with light, usually in the NIR range [15,16]. During treatment, the radiation that hits the surface of the photothermal agents is converted into heat, leading to an increase in temperature to a point that can initiate cellular apoptosis or lead to tumor cell necrosis [17,18,19]. Necrosis is considered the most probable primary cause of cell death through PTT. Especially when extremely high temperatures are achieved, this pathway corresponds to an early cell death caused exclusively by external factors [20,21]. The downside of necrosis lies in the fact that, as cell functions and structures abruptly break down, the cellular contents leak into the extracellular space, inducing inflammation. Also, in the later stages of necrosis, the remaining cells release pro-inflammatory factors, exacerbating the inflammatory response [20]. The following inflammation may lead to tumor recurrence and increased resistance to further therapies [22,23].

To perform PTT more effectively and adjust doses to avoid necrosis, photothermal agents with strong NIR absorption are necessary. Transition metal dichalcogenides (TMDCs) and other 2D nanomaterials have a high surface-to-volume ratio and great optical properties, namely strong NIR absorption and high photothermal conversion efficiency [24,25,26]. The optical and electronic properties of TMDCs are related to a band gap that increases as the lateral size and the number of layers decrease [27]. In addition, the absence of dangling surface bonds is responsible for their high stability in liquids [24]. The structure of TMDCs consists of a layer of transition metal atoms sandwiched between two layers of chalcogen atoms [28,29]. Usually, TMDCs for biomedical applications are prepared by top-down methods, which are also used for photoelectric devices and catalysis [30]. Molybdenum disulfide (MoS_2_)-based nanomaterials have great potential to be utilized as a platform for numerous biomedical applications and therapeutic approaches, such as photothermal and photodynamic therapy, imaging, drug delivery, and biosensing [31,32,33,34]. The crystal structure of MoS_2_ takes the form of a hexagonal plane of S atoms on both sides of a plane of Mo atoms [31]. These triple planes stack on top of each other, with strong covalent bonds between the Mo and S atoms but weak van der Waals forces holding the layers together [31,35]. As a result, they can be easily exfoliated using top-down methods based on mechanical and chemical approaches [31,36,37]. Single-layered or few-layered MoS_2_ nanosheets exhibit high absorption of near-infrared radiation (NIR) and high photothermal conversion efficiency [38]. In addition, this nanomaterial exhibits crystal-dependent fluorescence or fluorescence-quenching properties [31]. MoS_2_ nanostructures, especially MoS_2_ nanosheets, have also shown high stability, biocompatibility, high binding affinity to biomolecules, a large surface area, and remarkable magnetic and electronic properties [39,40]. Nanomaterials with a smaller lateral size than 100–200 nm and low-to-single-layer thickness are the gold standard for numerous biomedical applications, as the mentioned size and thickness promote biological interactions, the penetration of tissue and cell membranes, an improved permeability and retention effect (EPR), and rapid biodegradation and elimination [41,42]. Compared with other commonly studied photothermal therapy agents, such as metals, MoS_2_ presents relevant advantages, since metals are toxic in high concentrations and lack water stability. Furthermore, MoS_2_’s lateral size and layer number can be reduced to maximize its photothermal conversion to values above those achieved by metals [24,43].

Two-dimensional nanomaterial (2DnMat) conjugates provide excellent nanoplatforms for various synergistic therapeutic approaches, including the combination of PTT and chemotherapy. The best results in terms of treatment efficacy have been achieved with combined therapies [24]. Numerous papers have been published that confirm that the conjugation of 2DnMats with anticancer drugs significantly increase the efficiency of the treatments [44,45,46,47,48,49,50,51,52,53,54,55,56,57,58,59,60,61]. An example of a drug never tested for combined therapy, but that has great potential, is Tegafur (C_8_H_9_FN_2_O_3_), an FDA- and EMA-approved anticancer drug used to treat gastric and colorectal cancer [62,63,64]. Other tumors treated with this drug include skin, breast, and pancreatic cancer [65]. Tegafur is an inactive oral prodrug that is metabolized to 5-FU [66,67]. Its mechanism of action is the inhibition of the enzyme thymidylate synthetase, which leads to a definitive alteration of the DNA replication pathway [68]. A practical way to combine 2DnMats and drugs such as Tegafur is by incorporating them into pharmaceutical formulations. Hydrogel delivery systems can provide a controlled release of various therapeutic agents without significant toxicity [69,70]. Carbopol hydrogels are made of an acrylic polymer approved by the FDA, being an ingredient in various pharmaceutical formulations, used for topical administration through the skin. They enable controlled release, improve skin permeability, and exhibit high biocompatibility and thermal stability [71,72].

Here, a new pharmaceutical formulation of a Carbopol hydrogel containing MoS_2_ nanosheets and Tegafur was developed for combined phototherapy and chemotherapy of skin cancer. All conditions that are important for an effective and selective treatment are optimized, starting with the reduction in the MoS_2_ particle size to below 200 nm, through the ideal MoS_2_ and Tegafur concentration and irradiation time, which we determined using our custom-built NIR LED systems.

## 2. Materials and Methods

### 2.1. MoS_2_ Nanosheet Production

Molybdenum disulfide (MoS_2_) nanosheets were prepared by MoS_2_ 90 nm nanopowder (Merk, Darmstadt Germany) liquid-phase exfoliation and intercalation using polyvinylpyrrolidone (PVP) [73]. First, 2.5 g of PVP was dispersed in 1000 mL of deionized water; then, the dispersion was magnetically stirred for 1 h and mechanically stirred for 20 h (ATM40-3LCD, Ovan, Barcelona, Spain). The resulting dispersion was processed for 6 h in a custom-built recirculation system using a peristaltic pump (Watson Marlow 323 Peristaltic Pump, Falmouth, UK) and an industrial ultrasonic probe (Hielscher Ultrasonics GmbH, Teltow, Germany). The temperature was kept below 40 °C by a cooling bath (Julabo F12, Julabo GmbH, Seelbach, Germany). Finally, the washing process was completed by centrifuging three times at 8000 rpm [74].

### 2.2. MoS_2_ Nanosheets Characterization

#### 2.2.1. Transmission Electron Microscopy

The morphology and lateral dimensions of the aqueous MoS_2_ dispersions were analyzed by transmission electron microscopy (TEM, JEOL JEM 1400 TEM, Tokyo, Japan) at a concentration of 30 µg/mL. Samples were sonicated and a volume of 10 μL was applied to a carbon-coated TEM grid, where it was allowed to settle for 30 min. The lateral dimensions of MoS_2_ were measured using the ImageJ 1.53a software, with 80–588 counts per sample.

#### 2.2.2. Zeta Potential Measurements

MoS_2_ particles at a concentration of 10 μg/mL were analyzed using a Zetasizer Nano-NS (Malvern Instruments, Malvern, UK) in a disposable Zetasizer cuvette. Four measurements were performed at neutral pH and room temperature.

#### 2.2.3. Thermogravimetric Analysis

A thermogravimetric analysis (TGA) of the dehydrated MoS_2_ samples was performed using a Netzsch STA 449 F3 Jupiter instrument (Selb, Germany). The sample mass was between 6 and 8 mg. The thermograms were recorded between 30 and 1000 °C at a heating rate of 10 °C min^−1^ under nitrogen flow.

#### 2.2.4. Energy-Dispersive X-ray Spectrometry

MoS_2_ dispersions (30 µg/mL) were sonicated for 30 min, and a volume of 10 μL was applied to the surface of an aluminum-coated sample holder and allowed to dry overnight. EDS data were obtained using the EDAX Genesis X4M software (Oxford Instruments, Oxford, UK) after acquisition using a QUANTA 400 FEG-SEM (FEI, Hillsboro, OR, USA) with an accelerating voltage of 3 kV.

#### 2.2.5. UV–Visible Spectroscopy

Absorbance spectra of aqueous MoS_2_ dispersions with a concentration of 12.5 μg/mL were recorded with a Lambda 35 UV/vis spectrometer (Perkin-Elmer, Waltham, USA). The samples were placed in a 50 μL quartz cuvette (Hellma Analytics, Müllheim, Germany) with a light path length of 10 mm, and their spectra were recorded between 200 and 850 nm. Measurements were performed in triplicate at room temperature with a baseline correction based on water as a blank control.

#### 2.2.6. Photothermal Properties

To evaluate the light-to-heat conversion ability of MoS_2_ water dispersions and hydrogels, a volume of 600 µL was added to 48-well plates in both cases. All wells were irradiated with a custom-built LED-based system with NIR emission (810 nm) and an irradiance of 0.1 W/cm^2^ [75], measured using a Delta Ohm HD 2102.2 radiometer. In total, 24 LEDs (Model: WL-5P5050EP120IR-810 Lumixtar, Shenzhen, Guangdong, China) were used in a matrix configuration of 6 × 4, and each LED only directly illuminated one well. All of them were soldered to an individual aluminum star base of 16 mm and were dispersed in the top of an aluminum heatsink with 100 mm × 120 mm × 2 mm. Each LED had an epoxy resin lens creating a beam angle (2θ1/2) of 120°. An additional PMMA Lens was used on top of the LED lens to reduce the effective beam angle to 9°. The LEDs and LED parts were obtained directly from the mentioned manufacturer according to the author’s specifications.

The light-induced temperature rise of the samples was recorded for 30 min by placing a K-type thermocouple in the center and halfway up the suspension and connecting it to a TC-08 thermocouple data logger (Pico Technology, Eaton Socon, UK). Nine replicates per condition were performed, and the results are reported as the mean and standard deviation. Prior to irradiation, samples were pre-warmed to 37 °C in an incubator to replicate the conditions of the biological tests.

### 2.3. Hydrogel Production

Hydrogels (HGs) were prepared by dispersing Carbopol 974 NF (0.5% *w*/*v*) at a concentration of 5 mg/mL in water, MoS_2_, Tegafur, or MoS_2_ + Tegafur dispersions. The selected concentration of MoS_2_ was 125 µg/mL and that of Tegafur was 50 µg/mL. The dispersions were sonicated for 10 min, 1 h after preparation. Gelification was carried out by adding NaOH dropwise at an initial concentration of 0.5 M. Concentrations of 12.5 mM of NaOH were used for the dispersions of Carbopol and Tegafur, MoS_2_, and MoS_2_ + Tegafur.

### 2.4. In Vitro Studies

#### 2.4.1. Cell Culture

Biological studies were performed with A-431 human epidermoid carcinoma cells (ATCC, CRL-1555, Manassas, VA, USA) and HFF-1 human foreskin fibroblasts (ATCC, SCRC-1041, Manassas, VA, USA). Cells were cultured in Dulbecco’s Modified Eagle’s Medium (DMEM, Thermo Fisher Scientific, Waltham, MA, USA) supplemented with 10% (*v*/*v*) fetal bovine serum (Alfagene, Carcavelos, Portugal) and 1% (*v*/*v*) penicillin/streptomycin (Biowest, Pays De La Loire, France). Cells were maintained in a humidified atmosphere with 5% CO_2_ and 95% air at 37 °C. The medium was replaced every 2–3 days and the cells were detached when 80% confluency was reached. In biological experiments, the effect of Tegafur, MoS_2_ alone, and MoS_2_/Tegafur-loaded hydrogels was investigated with both healthy and cancer cells in the presence or absence of NIR irradiation, as described in detail below.

#### 2.4.2. Cytotoxicity Assays of Tegafur and MoS_2_ Nanosheets

A-431 or HFF-1 cells were seeded in 48-well plates at a density of 10,000 cells/well and 40,000 cells/well, respectively. After 24 h, the culture medium was replaced with MoS_2_ (125–500 µg/mL) or Tegafur dispersions (0.1–500 µg/mL) in complete DMEM and incubated for additional 24, 48, or 72 h. In brief, material dispersions were removed and cells were incubated in a 10% (*v*/*v*) resazurin reagent (Sigma-Aldrich, St. Louis, MO, USA) in complete DMEM at 37 °C and 5% CO_2_ for 2 h. The fluorescence of the supernatant (λ_ex/em_ = 530/590 nm) was measured using a microplate reader spectrophotometer (Synergy Mx, Bio-Tek Instruments, Winooski, VT, USA). Negative and positive controls for cell viability decreases were performed by incubating the cells in complete DMEM or with 10% (*v*/*v*) dimethyl sulfoxide (DMSO) in complete DMEM, respectively. Data for each sample were normalized to the negative control for cell viability decrease and the results are expressed as the mean percentage of the control and standard deviation. All assays were performed in triplicate with six replicates for each condition tested. These tests yielded the appropriate concentrations of Tegafur and MoS_2_ to be added to the HG for the next biological assays.

#### 2.4.3. Cytocompatibility of MoS_2_/Tegafur Hydrogels and Photothermal Therapy

A-431 or HFF-1 cells were incubated with HGs at a final concentration of 125 µg/mL of MoS_2_ and 50 µg/mL of Tegafur in DMEM for 24–72 h. Resazurin assays were then performed as described above. Live/dead staining was performed after 72 h, as described below. After incubating the cells with HG for 30 min, irradiation was performed using our custom-made NIR LED systems as described above (Section 2.2.6). After 24, 48, and 72 h, the medium was removed and the resazurin assay was performed, as described above.

##### Live/Dead Assays

The live/dead assay with fluorescent labeling was performed to assess the viability of cells after treatment with irradiated or non-irradiated Carbopol/MoS_2_/Tegafur. Live cells were identified with Calcein AM (Invitrogen, ThermoFisher Scientific, Waltham, MA, USA), which penetrates the cell membrane and labels both the nucleus and the cytoplasm. Dead cells were stained with propidium iodide (PI; ThermoFisher Scientific, Waltham, MA, USA), which penetrates only the damaged cell membranes and stains the nucleus. Cells were seeded in 48-well plates. After the different treatments (24, 48, and 72 h), cells were washed with PBS and incubated for 20 min at 37 °C in the dark with a solution of PI and of Calcein AM in PBS at 2.0 μg/mL. Then, a PI solution of 1.0 μg/mL in PBS was added to each well. Images were acquired using the Operetta CLS High-Content Imager (Perkin Elmer, Waltham, MA, USA) and data were processed using the Harmony software 5.2.

#### 2.4.4. Statistical Analysis

A statistical analysis was performed using the GraphPad Prism software (version 8.4.2, San Diego, CA, USA). For parametric data, a one-way analysis of variance (ANOVA) with Tukey’s tests for multiple comparisons was performed. Differences between the experimental groups are considered significant if *p* < 0.05.

## 3. Results and Discussion

### 3.1. MoS_2_ Dispersions’ Physico-Chemical Characterization

**Figure 1:**
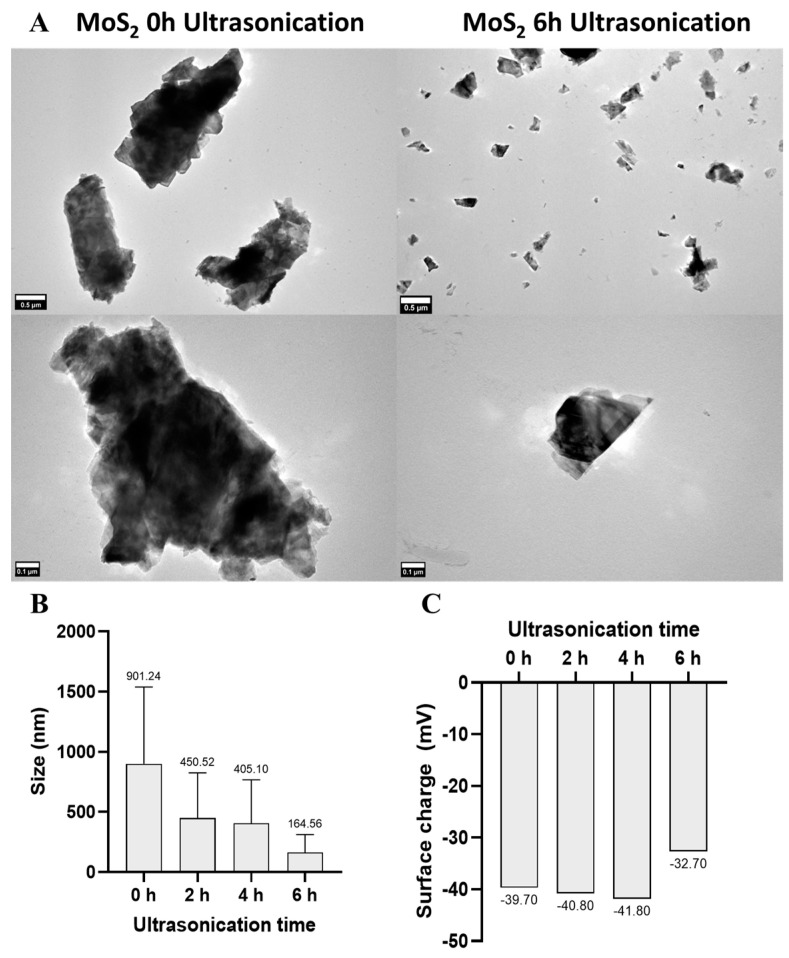
MoS_2_ particle size and morphology and surface charge at 0, 2, 4, and 6 h after ultrasonication. (**A**) Transmission electron microscopy (TEM) images of MoS2 aqueous dispersions at 0 and 6 h of ultrasonication (30 µg/mL). (**B**) MoS_2_ particle size after different ultrasonication periods, determined by TEM image analysis. (**C**) Surface charge of MoS_2_ particles (0, 2, 4, and 6 h of ultrasonication) determined by electrophoretic light scattering (ELS). (**A**) Scale bars represent 0.5 µm (**top images**) and 0.1 µm (**bottom images**).

Transmission electron microscopy (TEM) was used to investigate the particle size and morphology of the MoS_2_ nanosheets after fabrication and after ultrasonic treatment for different lengths of time in a custom-built recirculating system. The average size of the MoS_2_ nanosheets decreased from 901 ± 633 nm, before sonication, to 450 ± 372 nm, 405 ± 362 nm, and 165 ± 170 nm after 2, 4, and 6 h of sonication, respectively. The effectiveness of the ultrasonication method is shown by the fact that the lateral size of MoS_2_ decreases with the duration of sonication, while the degree of exfoliation increases. After 6 h of ultrasonication treatment, well-dispersed MoS_2_ layers were obtained without visible agglomeration, with good exfoliation and an average lateral size of 165 ± 170 nm (Figure 1). There is a substantial size heterogeneity in the samples, with the standard deviation decreasing as ultrasonication time increases (Figure 1B). After 6 h, we reach a size distribution ideal for the desired bioapplications in topical skin cancer phototherapy, since most particles present sizes in the range of 10–200 nm. Note that the particles present a few layers of thickness in the nanometric range at around 1–10 nm. This favors potential biological interactions, the penetration of tissue and cell membranes, improved permeability and retention effects (EPRs), and rapid biodegradation and elimination [41,42]. The zeta potential of the MoS_2_ nanosheets was determined by electrophoretic light scattering (ELS) using a Zetasizer device (1.C). The values were −39.7 ± 1.3 mV, −40.8 ± 1.3 mV, −41.8 ± 0.5 mV, and −32.7 ± 1.0 mV after 0, 2, 4, and 6 h of ultrasonication treatment, respectively. Particles with a surface charge below −30 mV are considered very stable and well dispersible in water [76]. This is consistent with the good stability and dispersibility of MoS_2_ nanosheets.

**Figure 2:**
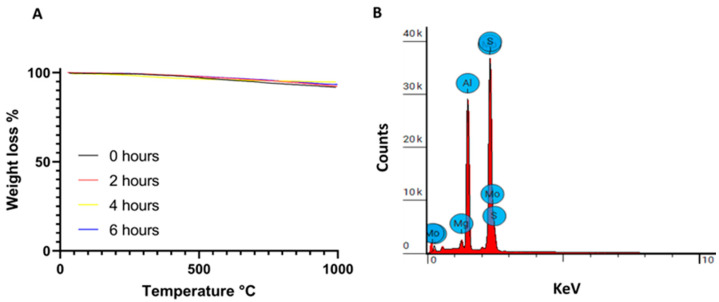
(**A**) Thermogravimetric analysis of MoS_2_ ultrasonicated for 0, 2, 4, and 6 h, performed under nitrogen atmosphere. (**B**) Energy-dispersive X-ray spectroscopy (EDS) analysis of MoS_2_ samples ultrasonicated for 6 h.

Thermogravimetric analysis (TGA) was used to evaluate the thermal stability of the MoS_2_ nanosheets after 0–6 h of ultrasonication. Figure 2A shows a gradual weight loss of about 7% in the tested temperature range. Other authors have observed similar behavior in thermogravimetric experiments with MoS_2_ under inert conditions, but no clear thermal degradation mechanism is suggested, aside from loss of chemisorbed water [77,78,79]. It was not possible to establish a correlation between the duration of ultrasonication treatment and the loss of thermal stability, as all samples showed only small weight losses, preserving more than 90% of the initial mass, indicating that the nanosheets produced have high thermal stability, even when the particle size decreases.

Energy-dispersive X-ray spectroscopy (EDS) was performed to determine the composition of MoS_2_ samples ultrasonicated for 6 h. Figure 2B and Table 1 reveal the presence of Mo, S, C, and Al and a vestigial presence of Mg. The presence of aluminum can be attributed to the sample holder in which the MoS_2_ samples were placed for analysis. The carbon comes from the PVP used in MoS_2_ production. Table 1 shows that the atomic ratio between S and Mo is about 1.8, which corresponds approximately to the expected theoretical ratio of two, as there must be two sulfur atoms to one Mo atom in each MoS_2_ molecule. Similar ratios have also been found in the literature [80].

**Figure 3:**
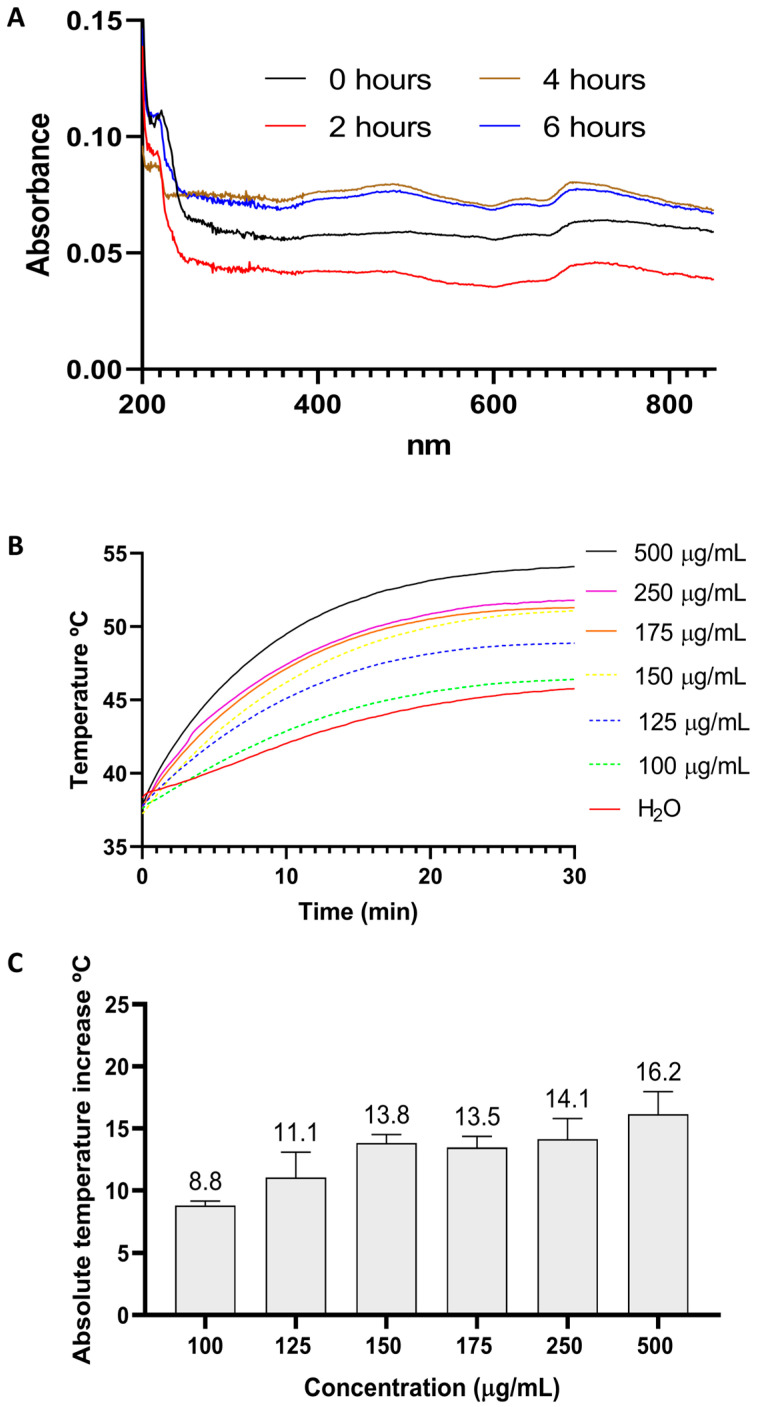
(**A**) UV–visible absorption spectra (200–850 nm) of MoS_2_ ultrasonicated for 0 h, 2 h, 4 h, and 6 h. (**B**) NIR light-to-heat conversion assays. Photothermal heating curves of MoS_2_ (6 h sonication) water dispersions (100–500 µg/mL) irradiated with a custom-built NIR LED system (810 nm, 0.1 W/cm^2^) for 30 min. Water was used as a control. (**C**) Absolute temperature increases of MoS_2_ (6 h sonication) water dispersions (100–500 µg/mL) irradiated with a custom-built NIR LED system (810 nm, 0.1 W/cm^2^) for 30 min.

The optical properties of MoS_2_ treated with ultrasound by ultrasonication for 0, 2, 4, and 6 h were investigated by UV-VIS spectroscopy (Figure 3A). The maximum absorption peaks for all samples are at 200 nm, which can be associated with the presence of PVP on the nanosheets, resultant from the production process [81]. Other peaks appear at 620 and 680 nm for all samples; according to the literature, these peaks can be accredited to the excitonic transitions from the K point to the Brillouin zone [82]. MoS_2_ sonicated for 6 h shows a 1.2-fold increase in NIR absorbance (810 nm) compared to the sample without ultrasonication. An increase in optical absorption with decreasing particle size and exfoliation is frequently observed in the literature for 2D nanomaterials [83,84]. Due to its smaller particle size and water stability, MoS_2_ ultrasonicated for 6 h was selected for the following assays.

To determine the potential of the six-hour-ultrasonicated MoS_2_ nanosheets as photothermal agents, aqueous dispersions of MoS_2_ (100 to 500 µg/mL) were irradiated with a custom-made LED-NIR device (810 nm, 0.1 W/cm^2^) for 30 min. The temperatures were registered at various time points, as illustrated in Figure 3B. After 30 min, 49, 51, and 54 °C were reached for MoS_2_ dispersions with the following concentrations: 125, 175, and 500 µg/mL.

### 3.2. MoS_2_ Cytocompatibility Optimization

**Figure 4:**
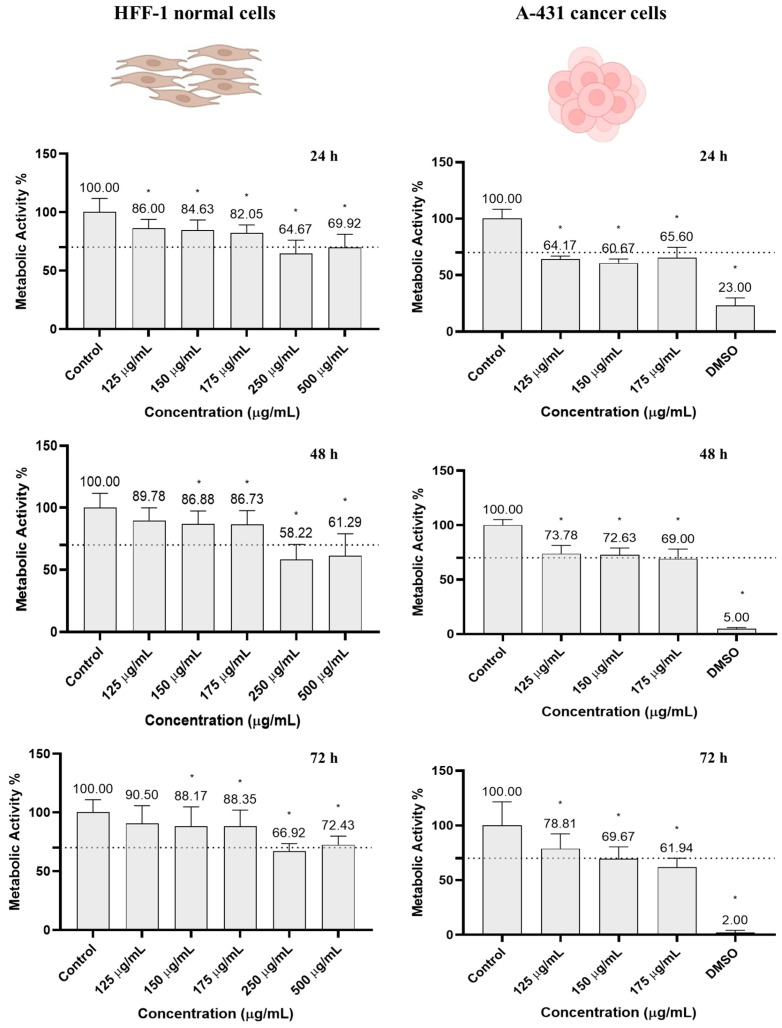
Metabolic activity of HFF-1 and A-431 cells incubated with MoS_2_ (125–500 µg/mL), determined using the resazurin assay after 24, 48, and 72 h. Results are normalized to values obtained for the control (complete DMEM). Results are presented as average and standard deviation (n = 6). Statistically significant differences against the control (complete DMEM) are represented as * *p* < 0.05. DMSO; dimethyl sulfoxide 10% (was used as positive control of cell death). The dashed line marks the toxicity limit (ISO 10993-5:2009 [86]).

Since MoS_2_ nanosheets have the potential to be used in various biomedical applications, including photothermal therapy, it is important to confirm that these nanoparticles are not toxic to healthy tissues [31,44,85]. For this reason, HFF-1 human fibroblasts were incubated with MoS_2_ (125–500 µg/mL) for 24, 48, and 72 h, as shown in Figure 4. At each time point, the cells’ metabolic activity was determined using the resazurin assay. Controls were performed with DMEM (cell-death-negative control, referred as “control”) and 10% DMSO (cell-death-positive control) only. 

Cells incubated with the two highest concentrations (250 and 500 µg/mL) showed a metabolic activity, already at the first time point (24 h), below the toxicity limit of 70% defined by ISO 10993-5:2009. Concentrations of 150 and 175 µg/mL led to a slight but significant decrease in metabolic activity of approximately 12% after 72 h. In contrast, no significant changes in metabolic activity were observed in cells incubated with 125 µg/mL after 72 h, as their metabolic activity was 90%. In view of the results described, concentrations below 175 µg/mL were considered safe for normal cells and, thus, this range was selected for further studies.

Next, to investigate the cytotoxic effect of the nanomaterial, MoS_2_ nanosheets were tested in A-431, a skin cancer cell line. Even without NIR irradiation, MoS_2_ was demonstrated to be cytotoxic. After 24 h, the cancer cell metabolic activity decreased to below 70%, but no cumulative cytotoxic effect was observed, as this activity remained at a similar level after 48 h and 72 h of incubation.

### 3.3. MoS_2_ Cytotoxicity under NIR Irradiation

**Figure 5:**
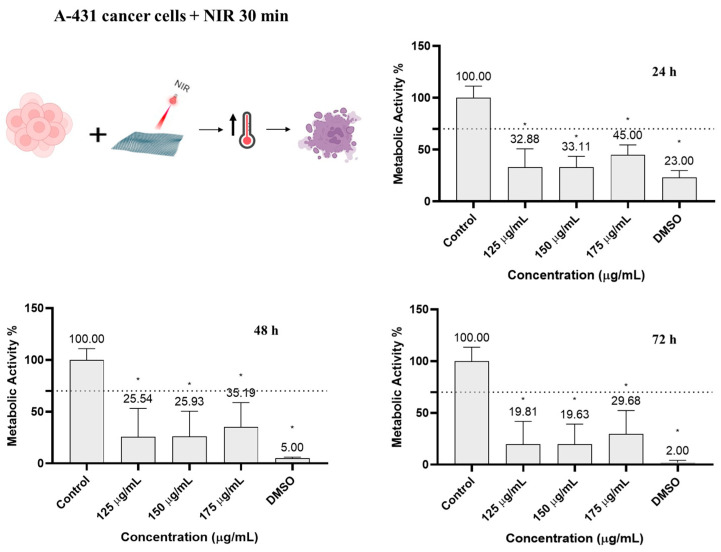
Metabolic activity of A-431 cells incubated with MoS_2_ and irradiated with NIR for 30 min (810 nm, 0.1 W/cm^2^, 180 J/cm^2^), determined using the resazurin assay after 24, 48, and 72 h. Results are normalized to values obtained for the control (complete DMEM). Results are presented as average and standard deviation (n = 6). Statistically significant differences against the control (complete DMEM) are represented as * *p* < 0.05. DMSO; dimethyl sulfoxide 10% (control of cell death). The dashed line marks the toxicity limit (ISO 10993-5:2009(E)).

To investigate the phototherapeutic effect of MoS_2_ nanoparticles under NIR irradiation, and to confirm that this effect is sufficient to kill A-431 skin carcinoma cells, they were incubated with different nanoparticle concentrations (125–175 µg/mL) and then irradiated for 30 min with a custom-built NIR LED system (810 nm, 0.1 W/cm^2^, 180 J/cm^2^). After 72 h, the metabolic activity of A-431 was approximately 20% at 125 µg/mL, 20% at a concentration of 150 µg/mL, and 30% at 175 µg/mL, proving that all tested concentrations had a significant photothermal effect on cancer cells after 30 min of irradiation (Figure 5). However, no significant differences in metabolic activity were observed between the groups of cells incubated at 125, 150, or 175 µg/mL, indicating that higher concentrations were not directly associated with a greater decrease in metabolic activity or a higher photothermal effect.

A concentration of 125 µg/mL was selected as the optimal concentration to be incorporated in the pharmaceutical formulations (Carbopol hydrogels) for further tests, as it reduced the metabolic activity of A-431 skin carcinoma cells, without affecting HFF-1 normal skin fibroblasts.

### 3.4. Tegafur Cytotoxicity/Cytocompatibility Optimization

**Figure 6:**
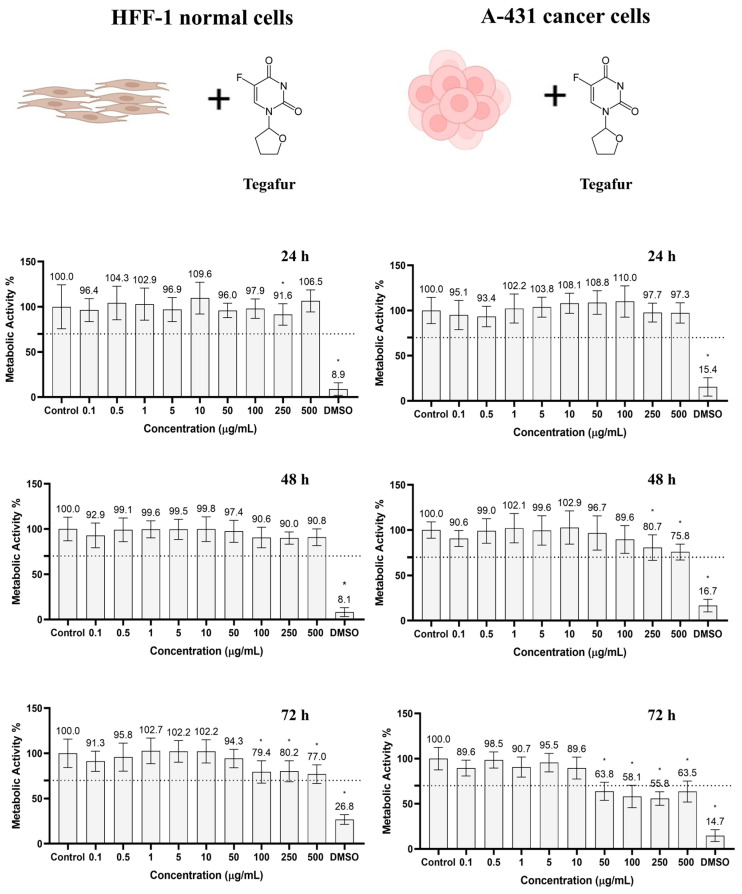
Metabolic activity of HFF-1 and A-431 cells incubated with Tegafur, determined using the resazurin assay after 24, 48, and 72 h. Results are normalized to the control (complete DMEM). Results are presented as average and standard deviation (n = 6). Statistically significant differences against the control (complete DMEM, negative control) are represented as * *p* < 0.05. DMSO; dimethyl sulfoxide 10% (positive control of cell death). The dashed line marks the toxicity limit (ISO 10993-5:2009(E)).

The cytotoxicity of Tegafur on human skin fibroblasts (HFF-1) and human skin carcinoma cells (A-431) was investigated to determine the minimum concentration that leads to cancer cell death without damaging healthy tissue. Both cell lines were incubated for 24, 48, and 72 h with different concentrations of Tegafur between 0.1 and 500 µg/mL. At each time point, the viability of the cells was tested using the resazurin assay (Figure 6). Controls were performed using cell culture media (DMEM, negative control) and DMSO 10% (cell-death-positive control) only.

At all tested concentrations and time points (24, 48, 72 h), HFF-1 showed a cell metabolic activity of more than 70% and was therefore considered as not cytotoxic, according to the ISO 10993-5:2009(E) norm. However, at concentrations of more than 50 µg/mL, the metabolic activity significantly decreased after 72 h of incubation.

In A-431 human skin carcinoma cells, metabolic activities below 70% were observed after 72 h at concentrations of Tegafur above 50 µg/mL. The metabolic activity values were 63.8, 58.1, 55.8, and 63.5% for 50, 100, 250, and 500 µg/mL, respectively. These results can be explained by the mechanism of action of Tegafur, which is metabolized into 5-FU, an anticancer drug that directly interferes with DNA replication. Since the mechanism depends on the metabolic activity of the cancer cells, it is expected that it takes some time for a sufficient concentration to accumulate in the cells, which eventually leads to a decrease in cell viability [66,68]. Since 50 µg/mL of Tegafur was biocompatible with HFF-1 human skin fibroblasts and toxic to A-431 human skin cancer cells, this concentration was chosen as the optimal amount for the pharmaceutical formulation with Carbopol, as described below.

## 4. Hydrogels’ Characterization

**Figure 7:**
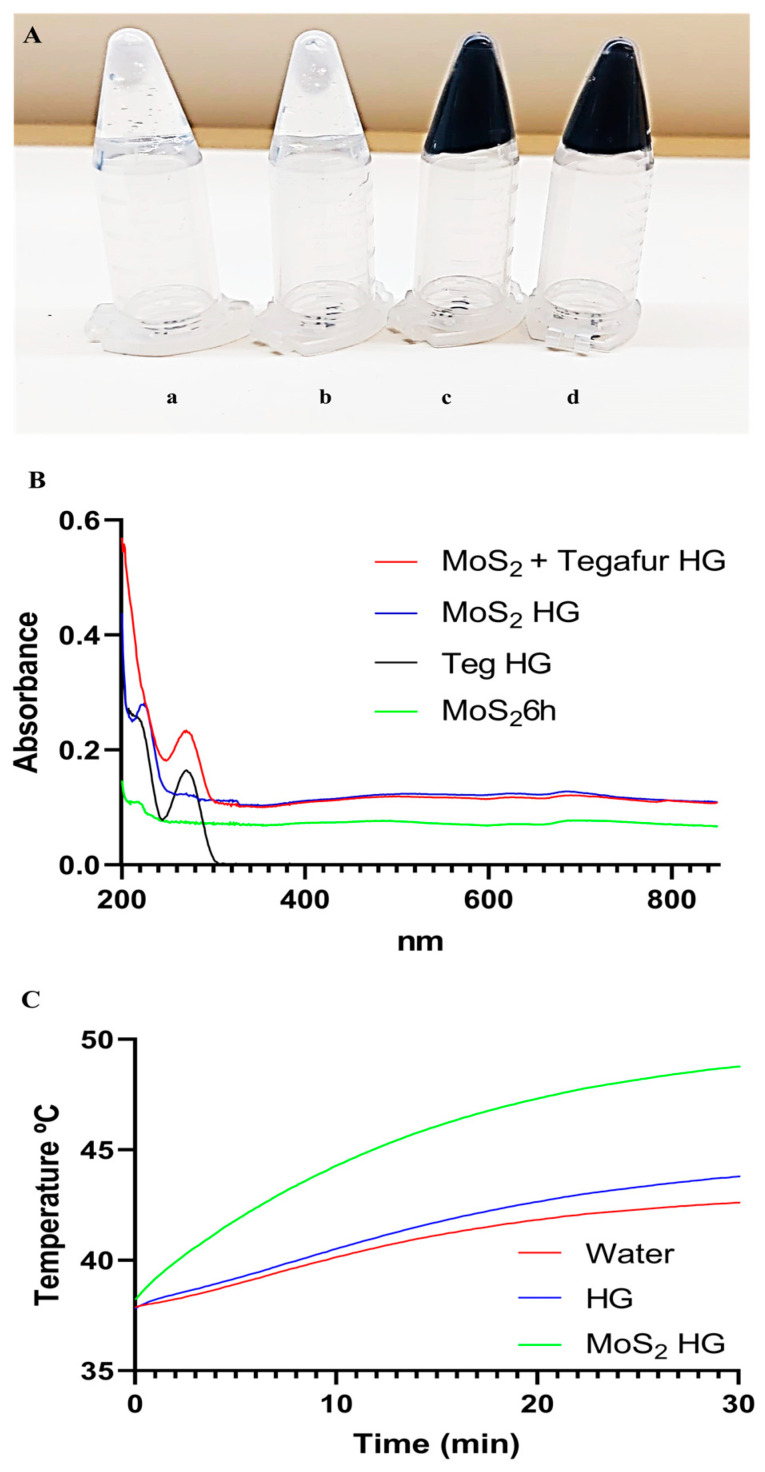
Hydrogels’ morphological and photothermal potential characterization. (**A**) Images of hydrogels right after being prepared in inverted Eppendorfs: (**a**) Carbopol hydrogel (0.5% *w*/*v*); (**b**) Carbopol/Teg hydrogel (0.050 µg/mL Tegafur); (**c**) Carbopol/MoS_2_ hydrogel (125 µg/mL MoS_2_); (**d**) Carbopol/MoS_2_/Teg hydrogel (125 µg/mL MoS_2_ + 50 µg/mL Tegafur). (**B**) UV-VIS absorption spectra (200–850 nm) of MoS_2_ + Tegafur HG, MoS_2_ HG, Tegafur HG, and a MoS_2_ dispersion ultrasonicated for 6 h. (**C**) Photothermal heating curves for Carbopol/MoS_2_ hydrogels. Water and Carbopol hydrogel were used as non-heating controls.

Carbopol hydrogels (HGs) were prepared by dispersing Carbopol 974 NF (0.5% *w*/*v*) at a concentration of 5 mg/mL in water, MoS_2_, Tegafur, or MoS_2_ + Tegafur water dispersions. The final concentration of MoS_2_ was 125 µg/mL and the final concentration of Tegafur was 50 µg/mL (based on previously described biological tests). Sodium hydroxide (NaOH) was then added to the solutions to allow gelification. The HGs containing MoS_2_ were black in color, while the HGs without MoS_2_ were completely transparent (Figure 7A).

Figure 7B shows the UV–visible absorbance spectra of MoS_2_ nanosheets in water dispersion, the MoS_2_ HG, the Teg HG, and the MoS_2_ + Teg HG. The maximum absorption peaks at 200 nm in all samples are due to the presence of PVP on the surface of the nanosheets [81]. Samples containing Tegafur show a typical absorption peak at 270 nm [87,88]. The NIR absorbance of the MoS_2_ + Teg HG and MoS_2_ HG (810 nm) increased 1.6-fold compared to the MoS_2_ 6 h ultrasonication water dispersions. To investigate the photothermal effect of the MoS_2_ nanosheets in the HG, NIR irradiation tests were performed. After 30 min of irradiation with a custom-made LED NIR device (810 nm, 0.1 W/cm^2^, 180 J/cm^2^), the HG containing MoS_2_ (125 µg/mL) reached approximately 49 °C. This temperature is within the range required for effective photothermal therapy of cancer (Figure 7C). Since Tegafur does not exhibit significant NIR absorption, the Carbopol/Teg HG was not tested.

### 4.1. MoS_2_/Tegafur Hydrogels’ Cytocompatibility

**Figure 8:**
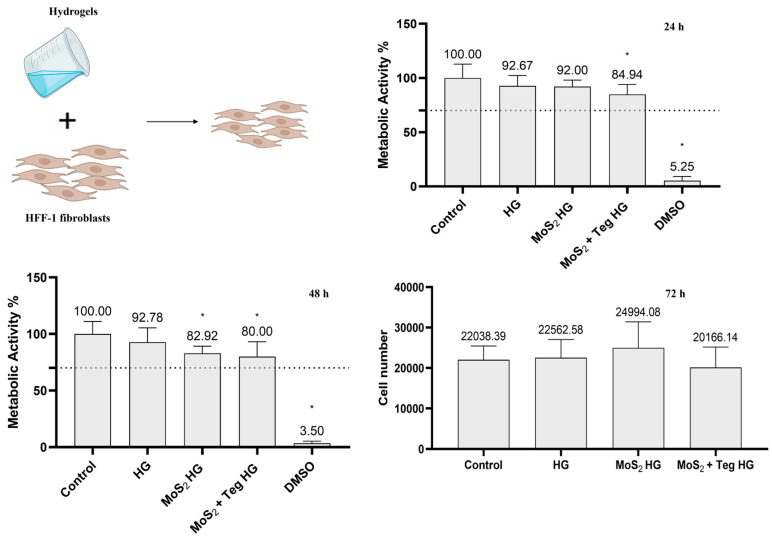
Metabolic activity of HFF-1 cells incubated with HG, HG containing MoS_2_, or MoS_2_ + Tegafur (Teg), determined using the resazurin assay after 24 h, 48 h, and 72 h. Results are normalized to values obtained for the control (complete DMEM). Results are presented as average and standard deviation (n = 6). Statistically significant differences against the control (complete DMEM, negative control) are presented as * *p* < 0.05. DMSO; dimethyl sulfoxide 10% (positive cell death control). The dashed line marks the toxicity limit (ISO 10993-5:2009(E)). Number of cells counted on the live/dead staining of HFF-1 cells incubated with HG or HG with MoS_2_ with and without Tegafur for 72 h. For all conditions tested, MoS_2_ was at a concentration of 125 µg/mL and Tegafur was at a concentration of 50 µg/mL. No statistically significant differences in the number of cells were found between all conditions tested.

HFF-1 skin fibroblasts were incubated for 24 and 48 h with the Carbopol HG dispersed in DMEM, the Carbopol/MoS_2_ HG (125 µg/mL), and with the HG containing both MoS_2_ (125 µg/mL) and Tegafur (50 µg/mL)—Carbopol/MoS_2_ + Teg. Controls were performed with cell culture media (DMEM) and DMSO 10% (cell death control) only. At each time point, the metabolic activity of the cells was determined using resazurin assays (Figure 8). At both time points, the metabolic activity was always above the toxicity limit of 70% specified in ISO 10993-5:2009(E). Therefore, neither MoS_2_ nor Tegafur caused toxicity to normal skin cells.

**Figure 9:**
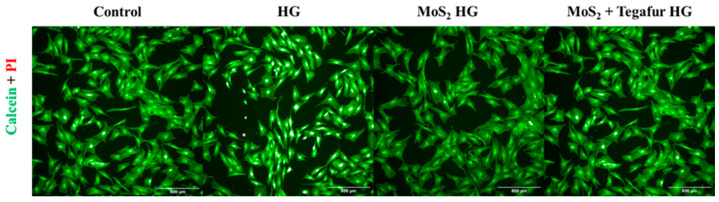
Live/dead staining of HFF-1 cells incubated with HG, MoS2 HG, and MoS_2_ + Tegafur HG for 72 h. MoS2 was at a concentration of 125 µg/mL and Tegafur was at a concentration of 50 µg/mL. The control corresponds to cells incubated with cell culture media (DMEM, negative control) only. Live cells metabolize calcein (green), and dead/dying cells are stained by propidium iodide (PI) (red), which penetrates their membrane. The scale bar represents 500 µm.

Live/dead staining of HFF-1 cells was performed to investigate the cytocompatibility of the HGs after 72 h of incubation. The number of cells/area was 22,039, 22,563, 24,995, and 20,166 for HFF-1 cells incubated with DMEM, Carbopol HGs, Carbopol/MoS_2_ HGs and Carbopol/MoS_2_/Teg HGs, respectively (Figure 8). Interestingly, no statistically significant differences in the number of cells were observed. Figure 9 also shows that the HFF-1 cells exhibited a normal spindle-shaped fibroblast morphology, metabolized calcein, and excluded propidium iodide under all conditions tested, confirming the cytocompatibility of the pharmaceutical formulation components.

### 4.2. MoS_2_/Tegafur Hydrogels’ Selective PTT Effect Optimization

Different irradiation times (15, 20, 25, and 30 min) were tested to determine the optimal condition to maximize the efficiency of the phototherapeutic effect. Tegafur is an inactive oral prodrug which is metabolized to 5-FU [66]. This inhibits the enzyme thymidylate synthetase, which will lead to a definitive change in the DNA replication mechanisms [68]. Tegafur should possess high targetability, since its activation requires a complex metabolic pathway that is more common in cancer cells [89].

Mild PTT strategies include a temperature increase to 39–45 °C, as cancer cells are less tolerant to heat stress; this therapeutic approach should present selectivity. The increase in temperature drastically reduces DNA and RNA synthesis, as well as DNA repair, and increases the permeability of tumor cells, leading to an increase in drug and nanomaterial intake [90,91]. The optimization of this effect should destroy the A-431 skin carcinoma cells without killing the normal HFF-1 skin fibroblasts. Therefore, both cell lines were incubated with the Carbopol HG containing MoS_2_ (125 µg/mL) and Tegafur (50 µg/mL). The cells were then irradiated with custom-made NIR LED systems (810 nm, 0.1 W/cm^2^) for 15, 20, 25, and 30 min. These irradiation conditions correspond to doses of 90, 120, 150, and 180 J/cm^2^.

Resazurin assays were performed to determine the effects of the treatment on the metabolic activity of the two cell lines after 24, 48, and 72 h.

**Figure 10:**
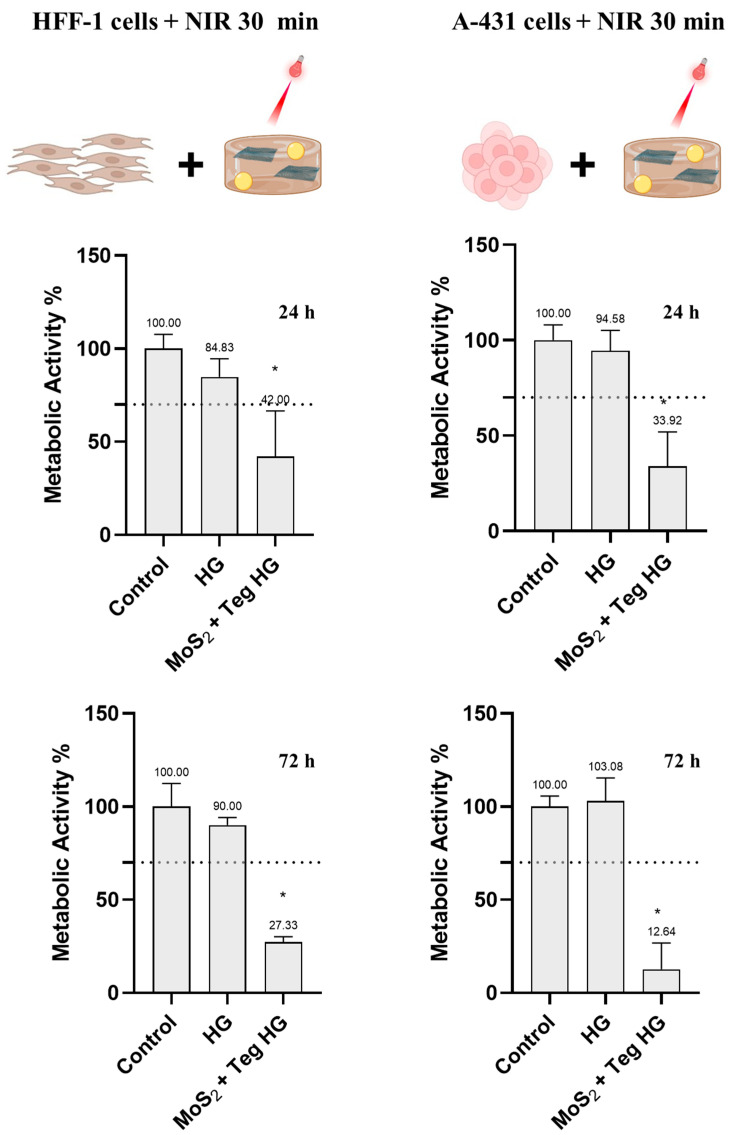
Metabolic activity of HFF-1 and A-431 cells incubated with MoS_2_ + Tegafur HG and irradiated for 30 min with NIR LED devices (810 nm, 0.1 W/cm^2^, 180 J/cm^2^), determined using the resazurin assay after 24 and 72 h. Results are normalized to values obtained for the negative control (complete DMEM). Results are presented as average and standard deviations (n = 6). Statistically significant differences against the control (complete DMEM) are presented as * *p* < 0.05. The dashed line marks the toxicity limit (ISO 10993-5:2009(E)). MoS2 was at a concentration of 125 µg/mL and Tegafur was at a concentration of 50 µg/mL.

First, both cell lines were incubated with the MoS_2_ + Teg HG and irradiated for 30 min (Figure 10). The resazurin tests show that the above conditions were highly cytotoxic for both cell lines at both tested time points (24 and 72 h). This is undesirable, as the treatment should be specific for skin cancer cells. Shorter irradiation times were therefore tested. 

**Figure 11:**
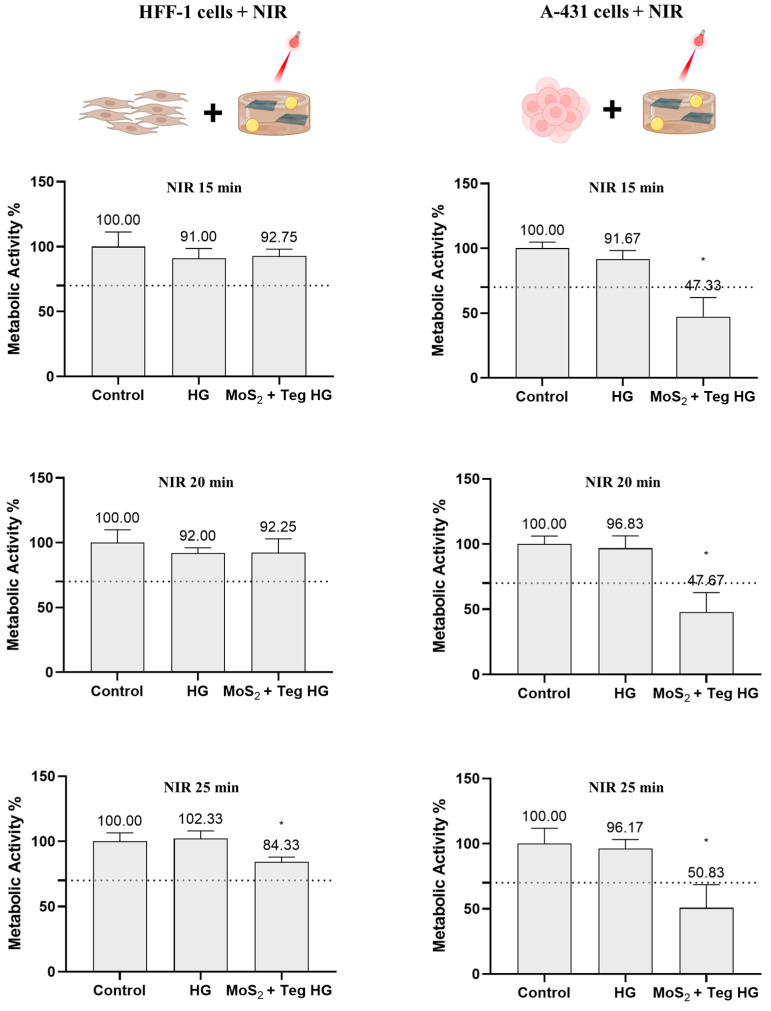
Metabolic activity of HFF-1 and A-431 cells incubated with MoS_2_ + Tegafur HG and irradiated for 15, 20, or 25 min with NIR LED devices (810 nm, 0.1 W/cm^2^), corresponding to doses of 90, 120, or 150 J/cm^2^, respectively, determined using the resazurin assay after 24 h. Results are normalized to values obtained for the negative control (complete DMEM). Results are presented as average and standard deviation (n = 6). Statistically significant differences against the negative control (complete DMEM) are presented as * *p* < 0.05. The dashed line marks the toxicity limit (ISO 10993-5:2009(E)). MoS_2_ was at a concentration of 125 µg/mL and Tegafur was at a concentration of 50 µg/mL.

HFF-1 cells irradiated with NIR for 15 and 20 min evidenced no cytotoxicity after 24 h and a metabolic activity of about 92%. After 25 min, the NIR irradiation decreased to 84%, which is still within the toxicity limit of 70% (ISO 10993-5:2009(E)) (Figure 11). In contrast, toxicity was observed in the A-431 skin carcinoma cells at all irradiation times tested, with metabolic activity values below 52% in all cases. No significant metabolic activity differences were found between the distinct irradiation times. For this reason, 15 min was chosen, as shorter irradiation times are most likely ensure cytocompatibility with the normal HFF-1 skin cells.

**Figure 12:**
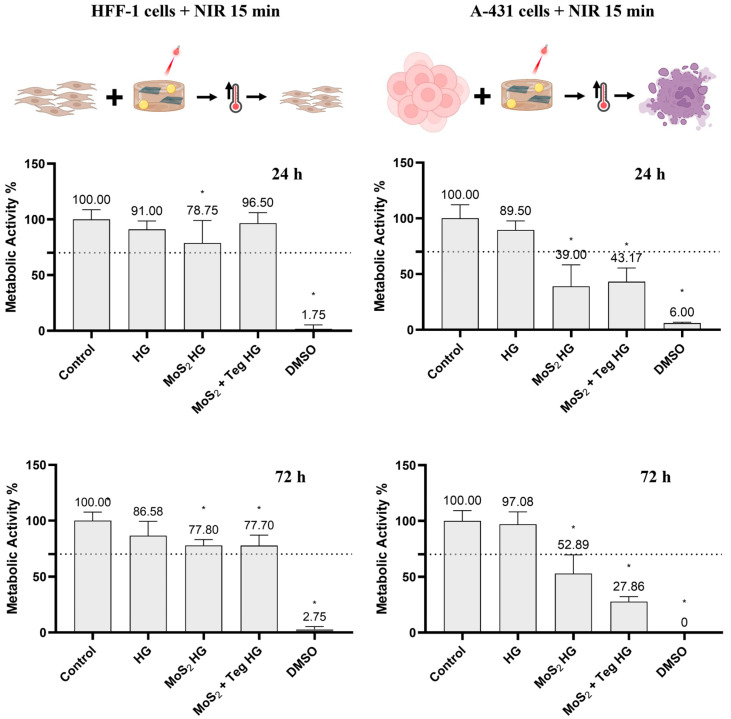
Metabolic activity of HFF-1 and A-431 cells incubated with MoS_2_ + Tegafur HG and irradiated for 15 min with NIR LED devices (810 nm, 0.1 W/cm^2^, 90 J/cm^2^), determined using the resazurin assay after 24 and 72 h. Results are normalized to values obtained for the negative control (complete DMEM). Results are presented as average and standard deviation (n = 6). Statistically significant differences against the negative control (complete DMEM) are presented as * *p* < 0.05. The dashed line marks the toxicity limit (ISO 10993-5:2009(E)). MoS_2_ was at a concentration of 125 µg/mL and Tegafur was at a concentration of 50 µg/mL.

Finally, both cell lines were treated with the pharmaceutical formulations and subjected to 15 min of NIR irradiation. Their metabolic activity was examined after 24 and 72 h (Figure 12). After the last time point (72 h), no cytotoxicity was observed for MoS_2_ or the MoS_2_ + Teg HG against normal HFF-1 skin cells, as the metabolic activity was about 78% in both cases. This value is above the cytotoxicity limit of 70% specified in ISO 10993-5:2009(E). In contrast, in the A-431 cells, the MoS_2_ + Teg HG clearly led to significant cytotoxicity after 72 h, as the metabolic activity was reduced to 28%. Moreover, this effect increased with time and was more pronounced in the presence of Tegafur than with the MoS_2_ HG alone, clearly indicating a combined effect between the anticancer drug and the nanomaterial.

Liu et al. demonstrated the selective photothermal effect of MnO_2_ functionalized with Au by incubating a human epithelial cell line (BEAS-2B) and a human lung adenocarcinoma cell line (A549) with the nanomaterial. After 24 h of incubation, both cell lines were irradiated with an 808 nm laser (1.5 W/cm^2^) for 10 min. A concentration of 50 µg/mL was able to reduce the viability of the A549 cancer cells to 13%, while the viability of the BEAS-2B normal cells was 75% [92]. Sahu et al. produced nano graphene oxide (nanoGO) non-covalently functionalized with Pluronic complexed with methylene blue (MB). HeLa cells and NIH/3T3 fibroblasts were incubated with the nanocomplex at a concentration of 10 µg/mL for 24 h. Subsequently, cells were irradiated with an 808 nm laser (2 W/cm^2^) for 3 min. The cell viabilities were approximately 70% and 40% for the NIH/3T3 fibroblasts and Hela cancer cells. The results revealed that the treatments achieved a selective effect [93]. Tegafur is an inactive oral prodrug which is metabolized to 5-FU [66]. 5-FU is also used for topical treatments of skin cancer and its mechanism is based on the inhibition of the enzyme thymidylate synthetase; the successful inhibition of this enzyme will lead to a definitive change in the DNA replication mechanisms [68]. Tegafur presents high selectivity since its activation requires a complex metabolic pathway, which includes steps that are 10 times more efficient in cancer cells than in normal cells [89]. Engel et al. studied the selectivity of Tegafur on normal astrocyte cells and human glioblastoma cells (U251). The cells were incubated with the anticancer drug for 72 h. The final half-maximal inhibitory concentrations (IC_50_) were 4295 and 384 µM for normal astrocytes and human glioblastoma cells, respectively. The results proved the high selectivity of this anticancer drug, as reported in our study (Figure 6) [94].

Mild PTT strategies involve increasing the temperature to between 39 and 45 °C, affecting mainly tumor cells, because they are less tolerant to heat stress. It inhibits DNA and RNA synthesis, as well as DNA repair, while tumor cell membranes become more permeable, improving drug and nanomaterial intake [90,91]. This strategy has been used to enhance chemotherapy effects. Above 60 °C, cell necrosis occurs through thermal ablation [90,91,95].

So far, authors have mostly used lasers that kill both normal and cancer cells. The use of LEDs presents several significant benefits when compared to laser sources, especially in applications requiring light irradiation. One of the foremost advantages is the cost-effectiveness of LEDs. On average, a high-powered NIR LED can be purchased for approximately EUR 20, a stark contrast to the cost of a similarly powered laser, which can range from several hundred to even thousands of euros. This makes LEDs an economically viable option for a wide range of applications. Moreover, the coherence of the light emitted by these two sources are fundamentally different and have practical implications. Laser light is highly coherent, meaning that it maintains a consistent phase across the beam, leading to a uniform wavefront. While this property is beneficial for certain applications, it poses challenges when irradiating heterogeneous semi-transparent materials, such as biological tissues. The high spatial coherence of laser light can result in the formation of interference patterns, leading to uneven energy distribution across the irradiated area. This unevenness can create high-intensity spots, which, in turn, can induce second-order effects (such as nonlinear optical phenomena) and localized thermal effects. These effects have the potential to cause localized damage to the material or sample being studied, which is particularly concerning in sensitive applications like medical treatments or biological research. In contrast, the light emitted by LEDs is inherently incoherent. This means that the phase of the light varies randomly across the beam, resulting in a more uniform irradiation pattern when the light interacts with these heterogeneous materials. The lack of coherence in LED light effectively mitigates the risk of forming high-intensity spots and the subsequent undesirable effects. Therefore, LEDs offer a safer and more controllable option for applications requiring gentle and uniform illumination, such as phototherapy [96,97].

To date, MoS_2_ selective LED-based phototherapy, together with combined chemotherapy, has never been shown in the literature. Zhang et al. irradiated MoS_2_ nanosheets functionalized with doxorubicin (DOX) with an NIR laser (808 nm, 1 W/cm^2^) for 15 min. After treatment, the viability of MDA-MB-231 breast cancer cells decreased to less than 20%, while the viability of L929, as normal mouse fibroblasts, decreased to less than 40% under the same conditions [48]. Yang et al. functionalized a MoS_2_ nanoparticle with melanin, hyaluronic acid, and DOX to perform combined chemo- and photothermal therapies. L929 cells and MCF-9 cancer cells were treated with 24 μg/mL of the nanocomplex and irradiated with an NIR laser (808 nm, 1 W/cm^2^) for 10 min. After treatment, the viability of MCF-7 breast cancer cells was reduced to about 20%. However, the viability of the L929 cells decreased to less than 40%, indicating high toxicity to healthy cells [47]. Therefore, the selectivity achieved in our study using MoS_2_ + Tegafur + LED NIR irradiation constitutes a novelty.

## 5. Conclusions

In this study, a new pharmaceutical formulation composed of a Carbopol hydrogel containing MoS_2_ nanosheets was proposed as an alternative innovative treatment to overcome the limitation of currently available therapies for skin cancer, such as invasiveness or a lack of selectivity.

After concentration versus cytotoxicity optimization, Carbopol pharmaceutical formulations were prepared containing 125 µg/mL of MoS_2_ and 50 µg/mL of Tegafur. An LED-based system emitting at 810 nm with an irradiance of 0.1 W/cm^2^ was shown to reduce skin cancer A-431 cells’ viability to 28%, after a treatment time of 15 min, corresponding to a dose of 90 J/cm^2^. Furthermore, a combined anticancer effect was identified when using MoS_2_ together with Tegafur. Also, no toxicity was found towards HFF-1 human skin fibroblasts.

In conclusion, this study demonstrated that MoS_2_ can be incorporated, together with Tegafur, in pharmaceutical hydrogel formulations, which, upon safe, LED-based NIR irradiation, leads to a combined destruction of skin cancer cells. This represents an innovative treatment strategy that enables safe and selective combined phototherapy and chemotherapy for skin cancer, constituting a possible alternative to currently offered therapeutic options.

## Figures and Tables

**Table 1:** Energy-dispersive X-ray spectroscopy analysis of MoS_2_ nanosheets after 6 h of ultrasonication.

Element Number	Element Symbol	Element Name	Atomic %	Weight %
6	C	Carbon	24.7	8.5
12	Mg	Magnesium	1.3	0.9
13	Al	Aluminum	32.1	24.8
16	S	Sulfur	27.0	24.8
42	Mo	Molybdenum	14.9	41.0

## Data Availability

The data presented in this study are available in this article.
